# Artificial Neural Network Models for the Prediction of Ammonia Concentrations in a Mediterranean Dairy Barn

**DOI:** 10.3390/ani15202967

**Published:** 2025-10-14

**Authors:** Luciano Manuel Santoro, Provvidenza Rita D’Urso, Claudia Arcidiacono, Fabio Massimo Frattale Mascioli, Salvatore Coco

**Affiliations:** 1 Building and Land Engineering Section, Department of Agriculture, Food and Environment (Di3A), University of Catania, 95123 Catania, Italy; luciano.santoro@phd.unict.it (L.M.S.); carcidi@unict.it (C.A.); 2 Electronics and Telecommunications, Department of Information Engineering, University of Rome “La Sapienza”, 00184 Rome, Italy; fabimassimo.frattalemascioli@uniroma1.it; 3 Section of Electrical, Electronics and Systems Engineering, Department of Electrical, Electronics and Informatics Engineering (DIEEI), University of Catania, 95123 Catania, Italy; salvatore.coco@dieei.unict.it

**Keywords:** artificial neural networks, livestock production, concentration, ammonia, dairy housing, multilayer perceptron, prediction

## Abstract

**Simple Summary:**

Ammonia (NH_3_) emission from dairy barns can negatively affect animal welfare, human health, and the environment. Predicting NH_3_ in real time is important for improving barn management and reducing pollution. In this study, artificial neural networks (ANNs) were tested to estimate NH_3_ concentrations in a Mediterranean dairy barn during different seasons. Environmental data, time of the day, and NH_3_ concentrations were applied to train and test three ANN models. The results showed high accuracy in predicting NH_3_ levels, demonstrating that this approach can facilitate Precision Livestock Farming (PLF) strategies, enhance livestock sustainability, and support animal welfare.

**Abstract:**

Understanding the relationship between environmental variables and gas concentrations from livestock production is essential for evaluating the impact of pollutants on animal housing and surrounding areas. This study investigates the use of ANNs to predict NH_3_ concentrations in a Mediterranean dairy barn under seasonal conditions—namely, hot, cold, and transitional weather. A Multi-Layer Perceptron (MLP) structure was employed, trained using Levenberg–Marquardt and Bayesian Regularization algorithms. The input dataset included ten variables related to internal and external environmental conditions, NH_3_ concentrations, and time of day. The models were evaluated using R^2^, R, MAE, MSE, and RMSE as performance metrics. Results showed strong predictive capabilities, with R^2^ values ranging from 0.75 to 0.96 and RMSE values between 0.47 and 0.80 due to the number of input data (different days) and environmental conditions. These findings highlight the potential of ANNs as effective tools for real-time pollutant prediction, supporting Precision Livestock Farming (PLF) strategies.

## 1. Introduction

Global livestock production is adapting to the increasing demand for commodities and foodstuffs, mainly due to population growth and wealth distribution influencing consumption patterns. In developed countries, while the demand for livestock products is steady, new approaches have aimed at reducing the impact of pollutants (e.g., ammonia (NH_3_), carbon dioxide (CO_2_), and methane (CH_4_)), and increasing efficiency and environmental sustainability [1,2]. Concentrations of pollutant gases constitute a major concern for the stakeholders and are the object of investigation in the scientific literature [3,4] due to the risk for human and animal health, disease spreading, environmental damages, and productivity reduction [5,6]. Although NH_3_ is not a greenhouse gas, its impact on the environment and health is well known and raises concern among farmers and researchers [7,8]. Since cows cannot fully utilize nitrogen obtained from the diet [9], a large portion of the NH_3_ is lost in livestock excreta, and such by-products cannot be easily stocked and managed, leading to dispersion in the environment [10]. In Europe, 75% of NH_3_ loss in the environment derives from livestock production [11]. In barns, gas concentrations can be monitored with various devices. High-precision methods, such as photoacoustic spectroscopy, provide accurate but costly results and require frequent maintenance, limiting their application. Conversely, low-cost sensors (i.e., optical absorption or metal oxide) are less accurate but more affordable, user-friendly, and portable, making them suitable for on-farm applications [12]. In addition, gas monitoring is influenced by seasonal variations, which affect gas dynamics and contribute to fluctuations in pollutant emissions [13].

Prediction models have been demonstrated to be a challenging but promising approach to evaluate milk yield [14], animal health [15], and gas concentrations [16,17]. New strategies emerged to build this kind of approach, and the most promising have been found to be artificial neural network (ANN) models [18,19]. ANNs are computational algorithms that mimic biological neural networks to make decisions, evaluate variables, and understand large datasets correlations [20]. These approaches have generally been demonstrated to have superior performance compared to traditional statistical methods [21,22].

ANN models have been implemented in prediction approaches to forecast mainly NH_3_ and CO_2_ concentrations from different livestock facilities in various countries. For instance, NH_3_ concentrations were predicted by applying Multilayer Perceptron (MLP) with environmental input data [23]; NH_3_ emissions were assessed through a Radial Basis Function (RBF) ANN model, involving directly measured input data from piggeries [24], while NH_3_ from livestock excreta was evaluated by using ANN models trained with manure characteristics derived from China’s Statistical Yearbooks [25]. CO_2_ concentrations have been the object of ANN approaches applied to a dairy barn, taking into consideration milk characteristics and age of calving and using MLP models for the trials [26], while MLP models were used in an investigation involving environmental data measured in piggeries [27]. Recent studies on NH_3_ and CO_2_ predictions have tested different training algorithms for MLP structures [23,26,27], often using the backpropagation algorithm, while others applied an RBF neural network in swine facilities [24]. However, all these articles lack any seasonal comparison between different datasets, and no evaluations have been carried out to assess the minimum amount of data required to make accurate predictions. Furthermore, in a previous study [28], authors highlighted a lack of reproducible methodologies in applying ANN models to predict emissions. Crucial information regarding ANN structures was often omitted by authors, especially key details for models (i.e., hidden layers and/or number of neurons), which were either partially reported or not disclosed at all.

On this basis, the objectives of this study were to (i) develop ANN models capable of predicting NH_3_ concentrations in a Mediterranean dairy barn under various seasonal conditions (“hot weather”, “cold weather” and “transitional weather”), (ii) assess how these models adapt when trained on distinct seasonal datasets, and (iii) evaluate their performance when using the complete yearly dataset. The models were trained by using the climate data and NH_3_ concentration collected for each seasonal period in order to identify the most suitable ANN configurations to achieve high prediction accuracy.

## 2. Materials and Methods

### 2.1. Barn Features and Timeframe of Investigation

**Figure 1:**
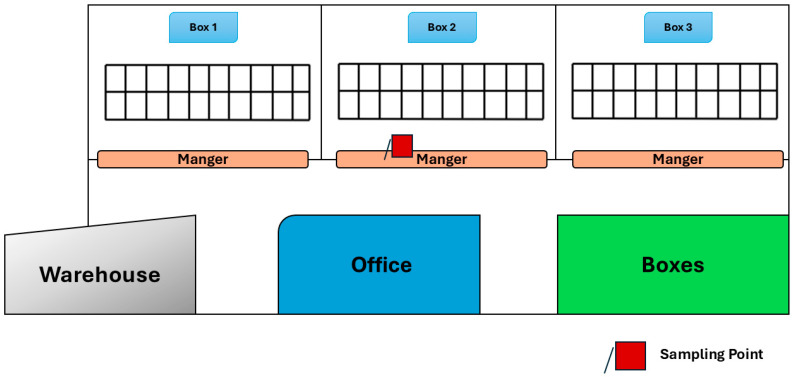
Planimetry of the barn under study with the sampling point selected.

All data were collected in a cubicle free-stall dairy barn (Figure 1) located in Vittoria (RG), Italy. The dairy house building, built with a steel and concrete structure and measuring approximately 55.50 m in length and 20.80 m in width, had three completely open sides (i.e., at the SE, NE, and NW), whereas the SW side was closed by a wall with four small openings, and a ridge vent at 7 m. The roof consisted of reinforced cement fiber panels. The natural ventilation system was integrated with two cooling systems located in both feeding and service alleys, mainly activated during hot and transitional seasons, when temperature thresholds were reached. In detail, below 20 °C, the fans were activated every 25 min for 4 min to promote air circulation. Ventilation was provided for 5 min every 15 min, starting from 18 °C in the feeding alley and from 20 °C in the resting area. The sprinklers were activated at temperatures above 26.6 °C. The barn had 60 head-to-head cubicles and was organized into three separate pens. Lactating Friesian-Holstein cows were milked twice per day in the early morning at 5:00 a.m. and in the afternoon at 5:00 p.m. in a separate milking parlour near the barn. The barn was cleaned once a day after the first milking at about 7:30 a.m. by using a mechanical tractor equipped with a scraper. After cleaning of the barn floor, a mixed concentrated feed, composed of insulate and forage, was delivered at about 11.30 a.m.

With a yearly average air temperature of 17.06 °C and a relative humidity of 73.54%, the climatic conditions in Vittoria (RG) are representative of a Mediterranean climate, classified as Csa in the Köppen classification [29]. The periods of investigation covered a whole year, from 17 September 2021 to 16 September 2022. The sampling point was located at the center of the barn, near the manger of box 2 (Figure 1).

### 2.2. Input Environmental Parameters and Output Gas Concentration Measurements

Climate and microclimatic parameters were measured using two weather stations (MeteoSense 2.0 GPRS + LAN, Netsens, Florence, Italy), installed inside and outside the barn and equipped with ultrasonic anemometers and thermo-hygrometers. These stations monitored and collected indoor and outdoor air temperature, air relative humidity, and wind speed and direction. Wind speed was measured with a resolution of 0.01 m/s, while temperature had an accuracy of ±0.15 °C. The internal station was placed inside the barn at 2.20 m above the central pen of the barn, whereas the external weather station was located at the ridge vent above the roof of the barn. Both weather stations operated continuously during the experiment, collecting data on the climatic variables considered every minute.

Concentrations of NH_3_ were continuously acquired using an INNOVA photo-acoustic analyzer composed of a Multigas Monitor mod. 1412i and a multipoint sampler 1409/12 (Lumasense Technology A/S). In this study, gas concentrations were recorded in a sampling point located in the central pen in the feeding alley at 40 cm from the barn floor. The selection of this sampling point was based on a previous study [30], which demonstrated that NH_3_ gas concentrations measured at various locations along the longitudinal axis of the barn differed significantly from those observed at other positions. The end of the sampling point tube was equipped with an air filter to keep the multipoint sampler free of particles. The INNOVA analyzer was calibrated by the device assistant just before the measurements. The setup of INNOVA had a sample integration time of 5 s. The accuracy of the Multi-Gas Analyser accounted for a 2–3% absolute deviation in concentrations, and the detection limit was 0.2 ppm for NH_3_.

### 2.3. Artificial Neural Network Models and Approaches for NH_3_ Prediction

ANN models used in this study were standard variants of MLP, created in MATLAB© by MathWorks. All experimentations were conducted in a machine with Microsoft Windows 10 Pro© 64 bit and the following specifications: Ryzen 7 5700 G (AMD, Dresden, Germany), 32.0 GB of RAM (Corsair, Fremont, CA, USA), and Radeon RX 7600 XT (AMD, Taipei, Tawain). MATLAB software versions were R2024a, R2024b, and R2025a.

**Figure 2:**
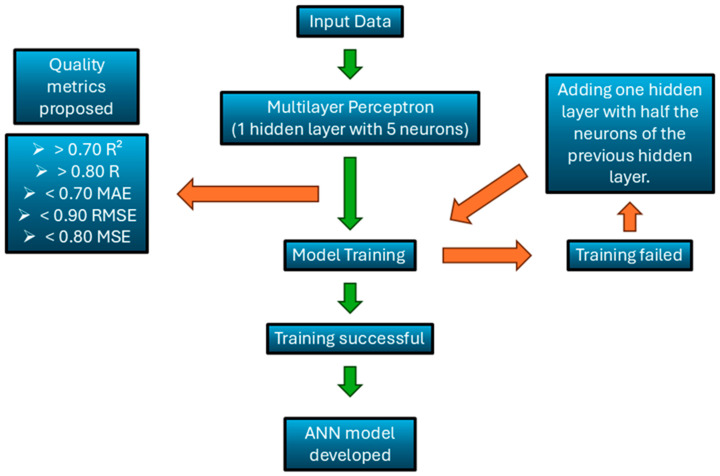
Flowchart of methodology applied in ANN construction.

In this study, the applied methodology is synthesized in Figure 2. In detail, the following subsections provide in-depth information on the dataset definitions, model architecture, training process, performance evaluation, and outlier management.

#### 2.3.1. Dataset Definition

The dataset was composed of 6480 records and included the day, hour of the day, indoor and outdoor air temperature, indoor and outdoor humidity, indoor and outdoor wind direction, and indoor and outdoor wind speed and NH_3_ concentrations. These parameters were acquired during the measurement campaign, and the mean hourly value of each parameter was considered for this study.

The main dataset was fractioned into three subsets: “hot” for summer (from June to September), “cold” for winter (from December to March), and “transition” (April, May, October, and November). This dataset subdivision is due to the dependence of NH_3_ concentration in naturally ventilated dairy barns on seasonal variations, as it was found in both scientific studies [13,31] and international guidelines (EEA/EMEP, 2019). Accordingly, subdividing the dataset by season allowed for a more robust estimation of NH_3_ concentration patterns, reducing the influence of the seasonal effects.

For each subset, a different number of days was selected: 2 days, 6 days, 30 days, and 60 days for each climatic period. In detail, for each climatic period, subdatasets with 2 days, 6 days, 30 days, and 90 days contained 48, 144, 720, and 2160 records, respectively.

#### 2.3.2. Model Architecture

In this research study, the most suitable ANN models for the specific application were studied. In this respect, we designed three ANN structures aimed at addressing specific tasks in livestock production. To evaluate the best ANN’s structure for each timeframe, a rationale was established: starting from the simplest structure, consisting of 1 hidden layer with only 5 neurons. This structure was used for the shortest input dataset (i.e., 6 days), and the performance was evaluated by using the evaluation metrics provided. Other tests were performed on this structure by adding more neurons to the layer, yet no significant improvement was observed in the results. To treat larger datasets (i.e., 12 days, etc.), 1 or more hidden layers were added to the initial structure. The number of neurons added in these hidden layers was chosen as half of the neurons of the preceding hidden layer. These models were evaluated by using established metrics and demonstrated the desired level of accuracy

#### 2.3.3. Training Process

Each model was retrained at least 3 times for the smaller datasets (6 and 12 days) and 10 times for the bigger datasets (90 days and 180 days). Furthermore, to avoid model overfitting, training was interrupted when a performance loss occurred during validation checks; such an approach is called “early stopping” and was applied to avoid quality loss during ANN model training. This approach consists of stopping the training when the model fits the noise in the data, decreasing performance in the ongoing iterations [28]. Default MATLAB© parameters and hyperparameters (e.g., normalization approach, learning rate, training data selection, and activation functions) have been considered during the tests, with the exception of the training algorithms (Table 1). In detail, min/max normalization was applied to the input variables; the learning rate was set to 0.01; training data were divided into 70% for training, 15% for test, and 15% for validation; and activation functions were *tansig* for hidden layers, and *purelin* for the output layer. It is worth highlighting that there was no need to modify default MATLAB settings, as this configuration already yielded accurate results.

#### 2.3.4. Performance Evaluation

The chosen validation criteria were the coefficient of determination (R^2^), coefficient of correlation (R), Mean Absolute Error (MAE), Root Mean Square Error (RMSE), and Mean Square Error (MSE) were chosen as validation criteria with values above 0.70 (R^2^), and 0.80 (R), and values below 0.70 (MAE), 0.90 (RMSE), and 0.80 (MSE). Although Mean Squared Error (MSE) does not rely on predefined thresholds, it remains a valuable metric for assessing model performance [32].

For each ANN model, all input layers used 9 environmental variables (i.e., indoor and outdoor air temperature, humidity, wind direction, wind speed, and hour of the day) and 1 output layer (NH_3_). According to the specific dataset, the first model aimed to predict short-term datasets (i.e., 6 and 12 days). This model consisted of three layers: input, output, and hidden layers. This layer was filled with 5 neurons to assess the prediction required. The second model, according to the “90 days” datasets, was trained with 3 hidden layers, each filled with 20, 10, and 5 neurons, respectively. The last model, according to the “180 days” dataset, was structured with 3 hidden layers, each filled with 40, 20, and 10 neurons, respectively.

In all ANN models, the learning process proceeded from the input to the output layers as a typical feedforward approach [33,34]. These models consisted of neurons and three types of layers: input, hidden, and output. In detail, input layers computed the entering variables, hidden layers performed the extraction and the transformation of the variables through a series of functions and weighted connections, and output layers produced the obtained predictions. Since MLP models are susceptible to overfitting [35], ANN models were trained with the Levenberg–Marquardt (LM) algorithm, and then the Bayesian Regularization (BR) algorithm was applied to reduce such inconsistencies. The BR algorithm introduced an extra parameter, called weight decay; this parameter decreases the variance of the weights to increase the robustness of the model [36].

#### 2.3.5. Outlier Management

In the dataset applied, several outliers were encountered during the test conducted. Outliers are records that strongly deviate from the other records collected. Such deviance can be generated by inadequate devices’ calibration and/or malfunctions, environmental anomalies, human and systemic errors, and inconstancies in recording procedures [37,38]. ANN’s performances can be negatively affected by the presence of such outliers. To avoid such negative effects in ANN performances, the Clean Data application in MATLAB© was applied to fill outliers with the nearest values method. Variables (i.e., internal and external air temperature and humidity, internal air speed and direction, and wind direction and velocity) were treated with the Clean Outlier Data. Outliers have been identified by the moving median detection approach and applying a threshold of 3, while the moving window had a window length equal to 5, and the window type was centered. The detection algorithm used was as follows: for each data point *x_i_*, the algorithm computes the median of a centered moving window (*W_i_*, length = 5). The point is flagged as an outlier if the following condition is met:

$$\frac{\left|x_{i}-median\left(W_{i}\right)\right|}{median(|W_{i}-median(W_{i})|)}\;>\;3$$

where the denominator is the Median Absolute Deviation, providing a robust estimate of local variability. In addition, the choice to replace the outlier with the nearest value was based on the following remarks: replacing an outlier with the closest non-outlier value prevents the introduction of artificial smoothing, which could distort short-term variations that are relevant for the modeling of gas concentrations. Furthermore, since large deviations are unlikely to represent real environmental changes but rather sensor noise or malfunction, replacing them with the nearest valid observation ensures continuity while keeping the signal within realistic ranges.

Alternative replacements (e.g., interpolation or median substitution) were considered, but the nearest value method was preferred to minimize alterations to the original data structure and preserve the natural temporal evolution of the measurements.

In detail, the number of outliers for each subdataset is reported in Table 2.

#### 2.3.6. Application of the Models

The three models were tested with the different datasets (e.g., 6 days, 12 days, 90 days, and 180 days) with and without outliers, for each subset (“hot weather”, “cold weather”, and “transition weather”). Furthermore, tests were carried out to assess the prediction ability of models trained with all the subsets. In addition, the performance was assessed in the presence and the absence of outliers.

## 3. Results

To assess the outcomes of the 3 MLP models, several tests were conducted by applying the ANN models to the different time-scale datasets. ANN structures were labeled with a numerical sequence that indicates the layers and neurons used (i.e., first the input layer, then the hidden layer with neurons, and lastly, the output layer). Therefore, the following simulations were carried out:

- MLP (1–5–1)—6 days, and 12 days for each dataset;
- MLP (1–20–10–5–1)—90 days for each dataset;
- MLP (1–40–20–10–1)—180 days for each dataset.

### 3.1. First MLP Model

All our first MLP models consisted of an input layer, an output layer, and a hidden layer with five neurons. The outcomes of our simulations are summarised in Table 3 for three different seasonal subsets (i.e., “hot weather”, “cold weather”, and “transitional weather”). In addition, in order to evaluate the impact of the outliers’ removal, other simulations were performed, and the results are shown in a specific raw data column.

As expected, in the results in Table 3, outliers have a significant impact on model outcomes. In particular, the presence of the outliers reduced the accuracy of all the models, with an accuracy reduction ranging from 0.02% to 0.15%. Interestingly, after outlier removal, the number of filled data points did not influence model outcomes. For this reason, an appropriate pre-processing methodology is needed to reduce the impact of outliers on the resulting accuracy.

**Figure 3:**
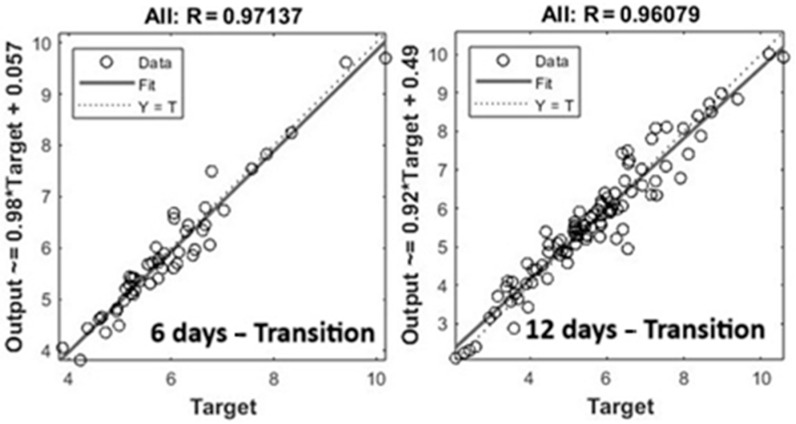
Linear regression of the best ANN model (i.e., 6-and 12-day transition) trained with filled data after outlier removal.

In order to evaluate the better-performing ANN models, we used linear regression plots. Linear regression results of the best ANN models obtained and trained with different datasets (i.e., 6 days and 12 days) are shown in Figure 3. In detail, in Figure 3, a linear regression model with filled data after outlier removal is presented. Results showed good agreement between fit and Y = T lines, confirming the prediction capacity of the models proposed. As expected, MLP models succeed in making accurate predictions, reasonably modeling the correlations between input variables analyzed and NH_3_ concentrations. In these trials, the transition season-based dataset achieved the best results.

### 3.2. Second MLP Model

All our second MLP models included an input layer, an output layer, and three hidden layers with 20, 10, and 5 neurons, respectively. The outcomes are summarized in Table 4 for three seasonal subsets (i.e., “hot weather”, “cold weather”, and “transitional weather”). To assess the impact of the outlier removal, additional simulations were conducted, and the corresponding results are reported in a raw data column in Table 4.

**Figure 4:**
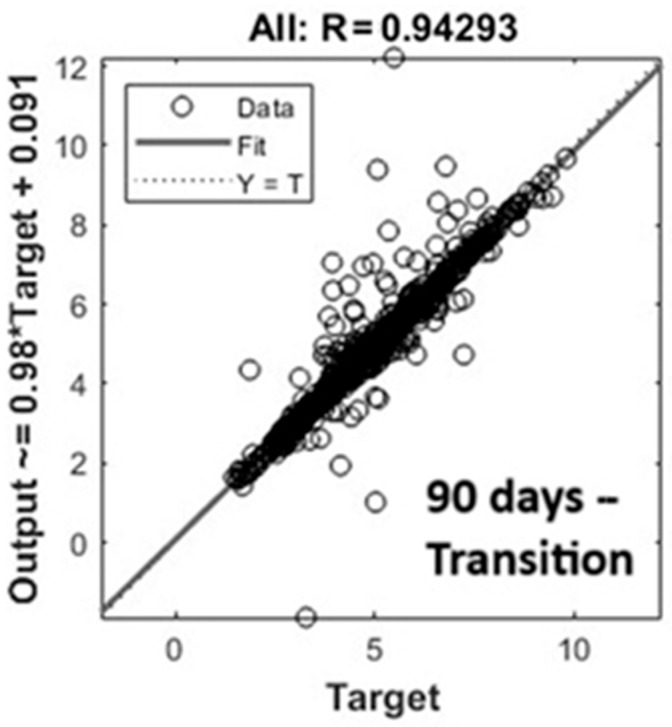
Linear regression of the best ANN model after 90 days—transition dataset without outliers.

Linear regressions are plotted in Figure 4 with the same graphical configuration as Figure 3. Fit lines were in good agreement with Y = T lines. However, the model underestimated the concentration of NH_3_, mainly for high values.

### 3.3. Third MLP Model

All our MLP models included an input layer, an output layer, and three hidden layers with 40, 20, and 10 neurons, respectively. In our simulations, the outcomes are reassumed in Table 4 across three different seasonal subsets (i.e., “hot weather”, “cold weather”, and “transitional weather”). Furthermore, to estimate the impact of outlier removal, additional simulations were carried out, and the results are displayed in a dedicated raw data column in Table 5.

**Figure 5:**
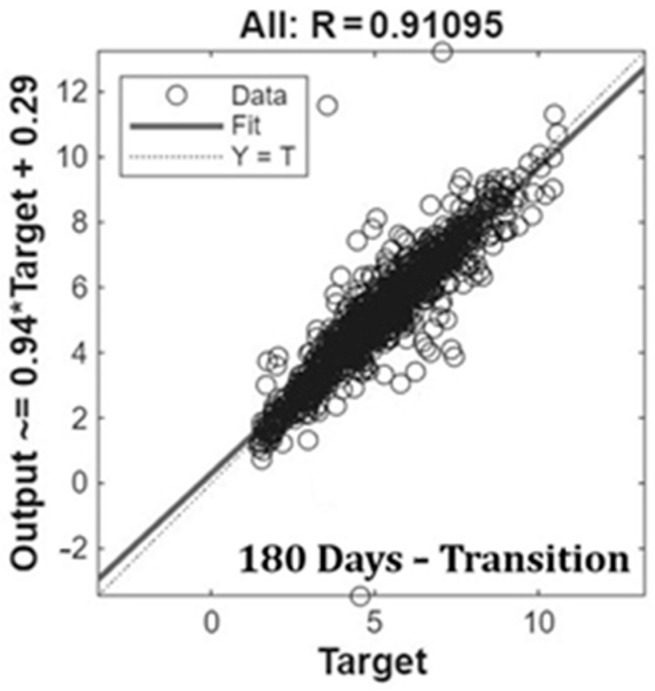
Linear regression of MLP models trained with the 180-day transition dataset.

The methodology applied was the same, training the model with the datasets with and without outliers (Figure 5). Linear regressions were in good agreement, according to the Fit and Y = T lines. However, the outcomes revealed a clear pattern of underestimation and overestimation, showing how data simulations related to very different climate conditions collected in a Mediterranean dairy barn were less accurate. Therefore, the choice to separately analyze data for different seasons was confirmed by the outcomes.

**Figure 6:**
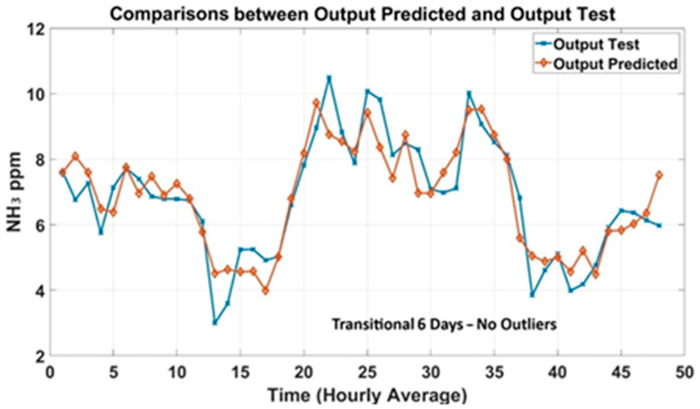
Prediction plots of 6-day transition dataset.

**Figure 7:**
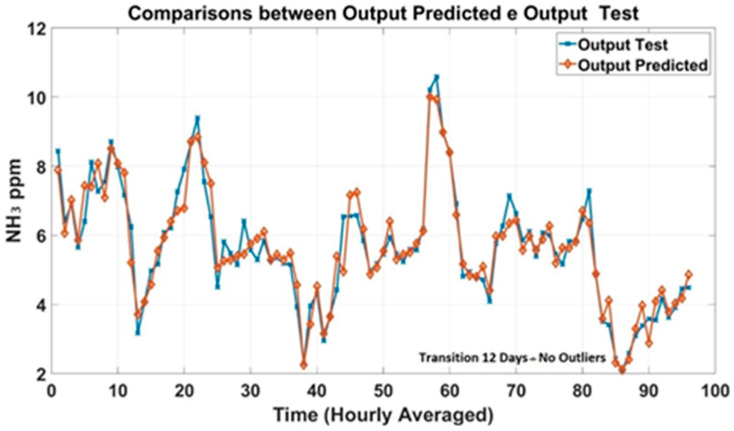
Prediction plots of the 12–day datasets for the transition season.

Prediction plots were generated to analyze the models’ trend based on historical data of NH_3_ concentrations collected in the barn. Figure 6 shows the predicted outcomes of the transitional 6-day dataset compared to the observed output (Test). The predicted NH_3_ concentrations were in good agreement with the measured concentrations. Prediction plots of the 12-day dataset were generated and shown in Figure 7 to evaluate the accuracy of the MLP models.

**Figure 8:**
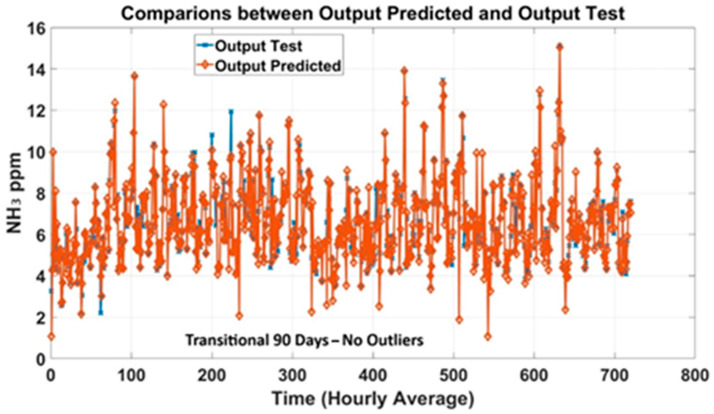
Prediction plots of the 90-day-based MLP models for each season (without outliers).

**Figure 9:**
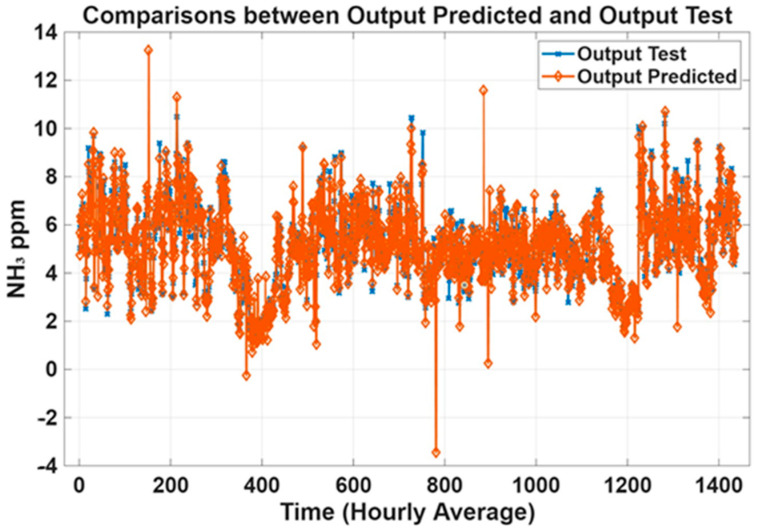
Prediction plots of MLP models trained with the 180-day datasets (without outliers).

For experimentations over 90 days, applying the same procedures, MLP models were trained on a dataset with raw data, including outliers, as well as on a dataset from which outliers were removed. Figure 8 exhibits predicted NH_3_ trends compared to NH_3_ concentrations measured in the barn. As expected, models trained without outliers (Figure 8) obtained better performance, confirming the MLP’s capacity to predict NH_3_ concentrations. The generated plot curves showed good alignment between the measured and predicted outputs. Figure 9 shows the curve trend of the MLP models trained with the data collected (without outliers) during the whole year. Analyzing the performance of the models, the curves’ trend highlighted acceptable predictions, aligned with the quality standards proposed. As a result, the performances were lower compared to the results achieved by the smaller dataset, but still acceptable for the purpose of this study.

Among the three seasonal scenarios, the “transition” period had the best performance, as shown in Table 1, Table 2 and Table 3. However, ANN performances for “hot weather” and “cold weather” scenarios were acceptable for the quality metrics, indicating ANN models’ capability to compute and generalize different datasets collected in different seasons. Notably, our ANN model trained with “hot weather” datasets encountered slightly more difficulties, compared to the transitional datasets, due to the higher gas variability during hot climatic conditions [39].

## 4. Discussion

The obtained results show the capability of ANN models to evaluate the concentrations of NH_3_ in a Mediterranean dairy barn. The selected input environmental variables were shown to be the most significant in the dynamics of the NH_3_ production. Indeed, environment variables have been widely investigated in the ANN literature, and their impact is well known in ANN applications.

The novelty of this approach lies in the detailed examination of all aspects involved in designing an ANN capable of providing accurate predictions of NH_3_ concentrations in the selected environment (i.e., the Mediterranean area). It is worth highlighting that the proposed ANNs have been tuned to address the specific task of NH_3_ prediction in the Mediterranean area, according to the available datasets, because the Mediterranean basin is considered a hot spot for studying climate change’s impact on the environment. However, this aspect does not affect the validity of the proposed neural approach since similar ANN models could be set up to predict other pollutant concentrations in different environments, provided the related datasets are available. In particular, the innovative contribution consists of identifying the minimum dataset required to ensure reliable predictions, as well as thoroughly assessing the validity of the time scale used. For this purpose, several different ANN models were created according to different time lengths of the collected data. In all cases, the model consistently achieved performance that was always higher than the threshold values. Notably, the time length of the dataset influenced the ANN performance; the best results were observed for the transitional dataset. In addition, our study on the influence of input dataset duration found that a minimum time span of 12 days was sufficient to achieve performance metrics comparable to or better than those obtained using datasets spanning 90 or 180 days. Moreover, we also compared the results obtained using several different transfer functions available in the MATLAB environment in ANN models. This is interesting because in the relevant livestock sector scientific literature, the behavior of several ANN models employing different transfer functions has not been investigated till now. It was found that the default MATLAB (i.e., tan-sigmoid) used for simulations shown in paragraph 3 obtained better results in comparison with simulations applying radial basis and log-sigmoid functions.

In this context, the outcomes of our study align with results appearing in several relevant publications. In a previous publication [40], the authors applied an approach in good agreement with this study, conducting tests with MLP and collecting environmental variables by monitoring devices and measuring NH_3_ with a photo-acoustic analyzer (i.e., Innova by Lumasense). Results align with our study, showing the robustness of MLP models in responding to this kind of challenge concerning livestock emissions. However, the authors collected data for a shorter period of time than our datasets, and the environmental conditions differed from the Mediterranean climatic conditions. Several MLP models with LM and BR training algorithms were applied, reporting RMSE values ranging from 0.83 to 96.26. However, this comparison with our simulations cannot be thoroughly exploited since in that work, ANN models were trained with input data obtained through infrared spectroscopy and assessed CH_4_ emissions involving animal-related variables (i.e., number of animals, age of calving, fat, milk, and protein yield) [26]. Another study [41] reported R^2^ values of 0.99 in assessing CO_2_ emissions from a poultry farm. ANN models were trained with LM and BR algorithms, while 1 hidden layer with several neurons ranging between 8 and 15 was applied. However, input variables consisted mostly of economic- and construction-related data (i.e., annual total savings, optimum insulation thickness, reduction in CO_2_, total wall heat resistance, insulation materials, fuels, interest rate, and building lifetime), while environmental data accounted only for 1 input variable (i.e., heating degree days). Conversely, in another study [18], authors carried out experiments based on the collection of zootechnical variables (i.e., mass of pigs, age, and feed intake). The metrics obtained aligned (i.e., R^2^ = 0.90) with the outcomes of our study. However, no information has been given regarding the models’ structures and hyperparameters.

A lack of information on ANN is generally indicated in other papers [18,23,42], where authors evaluated ANN performance in predicting CH_4_ concentrations in piggeries. Variables applied (i.e., CH_4_ concentrations and air temperatures) were measured directly in a timeframe aligned with this study; however, no information has been declared for ANN models, and the performance was worse compared to the metrics of this research. In a recent article [43], the authors evaluated the performance of different ANN models (i.e., Recurrent Neural Network and Compartmental Process-Based Neural Network) to predict NH_3_ emissions from dairy manure storage. Declared outcomes included an RMSE performance ranging from 1.64 to 8.06, which is slightly worse compared to our MLP models’ outcomes or other MLP models applied in similar studies [44,45]. Conversely, other authors applied several ANN [23] models (i.e., Backpropagation Neural Network, Recurrent Neural Network, and Particle Swarm Optimized Neural Networks) to estimate manure emissions. The outcomes align with the results of this study. However, the models proposed by the authors showed a significant reduction in accuracy in prediction with a longer time interval, reducing the application of their models to larger datasets. Remarkably, the authors omitted several key pieces of information regarding ANN models (i.e., training algorithms, overfitting management, and activation functions). Hyperparameters are crucial features in ANN models due to their importance in the training phase [46]. Among known hyperparameters, training algorithms are the most common due to their importance in the training steps. The Scale Conjugated Gradient (SCG) is a well-known algorithm able to train faster and converge on a large dataset [47]. Researchers have applied the SCG algorithm in ANN models to evaluate CO_2_ in poultry farms [42]. Results obtained suggested that SCG can be an asset to ANN training. However, from the tests conducted with this algorithm, an accuracy reduction was observed compared to the LM and BR algorithms applied in the same investigation.

Early stopping techniques have highlighted some difficulties in their applications [48], leaving as open questions “how” and “when” early stopping could be advantageously used. In our simulation, we applied early stopping during the training of our model, suitably modifying the available validation checks parameters of the MLP models in the MATLAB environment. To achieve this aim, some trials were carried out to assess the ANN model’s performance without any early stopping approach. In other trials, the restriction was removed. As expected, the trials implementing early stopping showed better performance with respect to ANN models trained without early stopping. It should be observed that our ANN models were adapted to the quantity of data in each considered dataset.

Such an adaptation could be chosen based on the data collection carried out and the devices involved. For example, ANN models with a simpler structure could be applied when data are acquired with low-cost sensors. These sensors are generally portable, user-friendly, affordable, and can be used by farmers without any particular training. Although low-cost devices, especially gas monitoring sensors, are inclined to saturate quickly, they can be applied to train ANN models with short datasets. In contrast, when larger datasets are available, ANN models can be more complex in order to provide more accurate predictions. In this case, ANN models could benefit from the use of high-cost devices. In fact, they determine gas concentrations with high reliability and are generally used in long-term monitoring, even though they are expensive and require training and maintenance to be used effectively [12]. In both cases, ANN can be a useful tool to predict NH_3_ using short datasets, reducing the effort required to acquire data in barns and lowering device cost. It is worth noticing that in all cases examined in our study, the observed prediction is always below the accepted values (i.e., 50 ppm of NH3 as the permissible exposure limit) for human and animal health, as reported by the Occupational Safety and Health Administration (OSHA) of the USA [49].

Therefore, traditional environmental monitoring strategies could be integrated with advanced strategies to mitigate these environmental impacts. Specifically, precision feeding automation, land use planning, water preservation, direct-use energy, ANN models, and advanced manure management can effectively support efforts to reduce environmental pollution [50]. These efforts are complemented by animal-related studies involving genetics and breeding research to reduce the contribution of enteric fermentation [51].

## 5. Conclusions

In this study, multiple ANN models were produced to predict NH_3_ concentrations in Mediterranean dairy barns, using seasonal datasets derived from three distinct climatic periods. After a suitable pre-processing phase, including the removal of outliers to ensure data reliability, the models demonstrated strong predictive performances. R^2^ values ranged from 0.75 to 0.96, while RMSE values were between 0.47 and 0.80, highlighting the robustness and accuracy of the developed models. These results confirm the potential of ANN-based approaches to be effective tools for real-time forecasting of pollutant dynamics in livestock environments. Beyond predictive accuracy, the findings also emphasize the role of ANNs in supporting sustainable barn management strategies, offering valuable insights for both environmental monitoring and decision making in the livestock sector, reducing the cost of computational analysis, and optimizing time-related applications of devices.

## Figures and Tables

**Table 1:** Training algorithms applied in the ANN models.

Training Algorithms	Description
Levemberg–Marquardt	The Levenberg–Marquardt algorithm is a fast and robust method for solving non-linear least squares problems, blending gradient descent with Gauss–Newton
Bayesian Regularization	Bayesian Regularization improves neural network generalization by penalizing complexity and preventing overfitting through a probabilistic framework

**Table 2:** Outliers removed from each dataset.

	Number of Outliers		
Subdataset	Hot	Cold	Transition
6 days	22	18	15
12 days	49	45	25
30 days	377	268	283
90 days	784	514	574

**Table 3:** Performance metrics in the different seasons.

	Hot Weather			
	6 days		12 days	
	Data without Outliers	Raw Data	Data without Outliers	Raw Data
R	0.92	0.84	0.92	0.85
R^2^	0.85	0.70	0.84	0.73
MAE	0.44	0.60	0.47	0.65
MSE	0.36	0.70	0.45	0.84
RMSE	0.60	0.83	0.67	0.92
	Cold Weather			
	6 days		12 days	
	Data without Outliers	Raw Data	Data without Outliers	Raw Data
R	0.93	0.87	0.96	0.92
R^2^	0.85	0.76	0.92	0.83
MAE	0.25	0.48	0.36	0.45
MSE	0.22	0.35	0.25	0.55
RMSE	0.47	0.59	0.50	0.74
	Transitional Weather			
	6 days		12 days	
	Data without Outliers	Raw Data	Data without Outliers	Raw Data
R	0.97	0.84	0.96	0.92
R^2^	0.92	0.70	0.93	0.85
MAE	0.36	0.75	0.36	0.39
MSE	0.25	0.94	0.22	0.43
RMSE	0.50	0.97	0.47	0.65

**Table 4:** Performance metrics in the different seasons.

	Hot Weather	
	90 days	
	Data without Outliers	Raw Data
R	0.93	0.88
R^2^	0.85	0.73
MAE	0.32	0.41
MSE	0.54	0.97
RMSE	0.73	0.98
	Cold Weather	
	90 days	
	Data without Outliers	Raw Data
R	0.93	0.85
R^2^	0.85	0.73
MAE	0.32	0.50
MSE	0.53	0.64
RMSE	0.72	0.80
	Transitional Weather	
	90 days	
	Data without Outliers	Raw Data
R	0.94	0.90
R^2^	0.88	0.80
MAE	0.24	0.32
MSE	0.27	0.48
RMSE	0.50	0.69

**Table 5:** Performance metrics in “180 days” dataset.

	Hot Weather	
	180 days	
	Data without Outliers	Raw Data
R	0.86	0.85
R^2^	0.71	0.70
MAE	0.52	0.60
MSE	0.80	0.78
RMSE	0.90	0.88
	Cold Weather	
	180 days	
	Data without Outliers	Raw Data
R	0.87	0.85
R^2^	0.72	0.70
MAE	0.50	0.40
MSE	0.76	0.80
RMSE	0.87	0.90
	Transitional Weather	
	180 days	
	Data without Outliers	Raw Data
R	0.91	0.89
R^2^	0.81	0.76
MAE	0.37	0.46
MSE	0.49	0.56
RMSE	0.70	0.76

## Data Availability

Data are available upon request to the corresponding author.

## Abbreviations

The following abbreviations are used in this manuscript:

NH_3_	Ammonia
ANN	Artificial Neural Network
PLF	Precision Livestock Farming
MLP	Multilayer Perceptron
CO_2_	Carbon Dioxide
CH_4_	Methane
RBF	Radial Basis Function
SE	Southeast
NE	Northeast
NW	Northwest
SW	Southwest
RG	Ragusa
R^2^	Coefficient of Determination
R	Coefficient of Correlation
MAE	Mean Absolute Error
RMSE	Round Mean Square Error
MSE	Mean Standard Error
LM	Levenberg–Marquardt
BR	Bayesian Regularization
